# Excess risk and resource utilization in dialysis-dependent patients undergoing total hip arthroplasty: insights from a nationally representative database

**DOI:** 10.3389/fsurg.2025.1704799

**Published:** 2026-01-16

**Authors:** David Maman, Yaniv Steinfeld, Yaron Berkovich

**Affiliations:** 1 Rappaport Faculty of Medicine, Technion University Hospital (Israel Institute of Technology), Haifa, Israel; 2 Department of Orthopedics, Carmel Medical Center, Haifa, Israel

**Keywords:** end-stage renal disease, healthcare costs, in-hospital mortality, length of stay, postoperative complications, renal dialysis, total hip arthroplasty

## Abstract

**Background:**

Total hip arthroplasty (THA) reliably restores function and quality of life in patients with end-stage hip disease. Individuals with end-stage renal disease (ESRD) requiring dialysis are vulnerable to perioperative complications due to immune dysfunction, hemodynamic instability, and impaired wound healing. Evidence on risk-adjusted outcomes remains limited.

**Methods:**

A retrospective cohort study was conducted using the Nationwide Inpatient Sample (2016–2022). Dialysis dependence was identified using ICD-10-CM Z99.2. Primary outcomes included in-hospital mortality, length of stay (LOS), and hospital charges; secondary outcomes were major complications. Propensity score matching (10:1 nearest neighbour) balanced baseline demographics and comorbidities. Survey weights were used for national estimates. Post-matching outcomes were compared using risk ratios (RR) with 95% CIs.

**Results:**

Among 1,957,284 THA patients, 2,730 (0.1%) were dialysis dependent. In unmatched analysis, dialysis dependence was associated with substantially increased in-hospital mortality (1.3% vs. 0.03%; crude RR: 31.9, 95% CI: 22.7–44.9). After 10:1 propensity-score matching, dialysis-dependent patients had 62.5% longer LOS (3.9 vs. 2.4 days, *p* < 0.01), 95.8% higher hospital charges ($98,454 vs. $60,741, *p* < 0.01), and persistently elevated in-hospital mortality (matched RR: 10.1, 95% CI: 6.3–16.2). Major complications were significantly more frequent.

**Conclusion:**

Dialysis dependence is a strong independent predictor of higher perioperative morbidity, mortality, and cost following THA. Targeted multidisciplinary optimization and enhanced perioperative care pathways are warranted.

## Introduction

Total hip arthroplasty (THA) is one of the most common and cost-effective surgical interventions for end-stage hip disease, consistently providing substantial pain relief and functional improvement (1). With an aging population and rising prevalence of degenerative joint disease, the number of primary THA procedures is projected to grow steadily worldwide over the coming decades (2). While perioperative complication rates for THA remain low in the general population, certain subgroups including patients with significant comorbidities are known to have disproportionately higher risks and resource utilization (3, 4).

End-stage renal disease affects patients across a wide age spectrum, though the burden increases markedly with advancing age. Recent epidemiologic reports show that dialysis dependence is most prevalent in older adults, who also represent a growing proportion of candidates for elective THA. Prior studies evaluating arthroplasty outcomes in dialysis-dependent patients although limited in size have consistently demonstrated elevated complication rates, higher perioperative mortality, and greater resource utilization compared with the general population. These findings underscore the importance of studying this high-risk group in a large, nationally representative cohort such as the NIS.

Patients with end-stage renal disease (ESRD) represent a particularly vulnerable population. Dialysis dependence is associated with profound alterations in immune function, bone metabolism, and hemostasis, which may predispose to infection, impaired wound healing, anemia, and cardiovascular instability. These physiological factors, combined with a higher baseline comorbidity burden, may lead to worse perioperative outcomes, longer hospitalizations, and higher costs following elective orthopedic procedures.

Despite these concerns, large-scale studies specifically examining the impact of renal dialysis dependence on THA outcomes remain limited. Most prior investigations have been single-center series or have combined THA and TKA into a single “arthroplasty” cohort, limiting the ability to draw THA-specific conclusions. Furthermore, many reports lack rigorous risk adjustment, leaving uncertainty regarding whether adverse outcomes are attributable to dialysis itself or to confounding patient characteristics (5)

The present study aimed to fill this knowledge gap by leveraging the Nationwide Inpatient Sample (NIS), the largest all-payer inpatient database in the United States, to examine outcomes of primary THA among patients with and without renal dialysis dependence (6). We sought to (1) compare patient demographics and comorbidities between dialysis and non-dialysis groups, (2) quantify differences in perioperative complications, length of stay, hospital charges, and in-hospital mortality, and (3) apply propensity score matching to generate well-balanced cohorts, providing an adjusted assessment of dialysis-specific risk (7).

## Materials and methods

### Data source

This study utilized the Nationwide Inpatient Sample (NIS), a stratified and weighted database within HCUP. NIS sampling weights, stratification variables, and clustering by hospital were applied according to HCUP recommendations to generate nationally representative estimates.

### Exposure definition

Renal dialysis dependence was defined using ICD-10-CM Z99.2. This code generally captures patients receiving chronic dialysis; however, the NIS does not differentiate dialysis modality, ESRD stage, or timing of treatments, and acute/temporary dialysis may be under-captured. This potential misclassification is acknowledged as a study limitation.

### Patient selection

We included all adult patients (≥18 years) who underwent elective primary total hip arthroplasty (THA) as identified by relevant ICD-10-PCS procedure codes. We excluded revision THA procedures, urgent or trauma-related admissions, and cases missing key demographic information. Discharges with COVID-19-related codes were omitted to avoid confounding from pandemic-related risk factors.

Renal dialysis dependence was defined by the ICD-10-CM diagnosis code Z99.2 (“Dependence on Renal Dialysis”). Patients were stratified into two cohorts based on dialysis status for comparative analysis.

### Variables and comorbidities

Baseline variables included demographic data (age, sex, race, and primary payer) and clinical comorbidities derived from ICD-10-CM codes. Comorbidities of interest included dyslipidemia, chronic anemia, osteoporosis, type 2 diabetes mellitus, congestive heart failure, chronic lung disease, liver disease, prior myocardial infarction, and prior cerebrovascular accident. Obesity was included as an additional risk factor.

### Endpoints

Our primary outcomes were:

- In-hospital mortality
- Length of stay (LOS), reported as mean ± standard deviation
- Total hospital charges, reported in U.S. dollars

Secondary outcomes consisted of major postoperative complications, including blood loss anemia, perioperative blood transfusion, pneumonia, prolonged mechanical ventilation (>24 h), deep vein thrombosis (DVT), and postoperative pain (ICD-10-CM G89.18). For each binary outcome, risk ratios (RR) with 95% confidence intervals (CI) were computed.

### Propensity score matching

Propensity scores were computed using logistic regression including demographics, comorbidities, and hospital characteristics (teaching status, region, and bed size). A 10:1 nearest-neighbour match without replacement was used. Standardized mean differences (SMDs) were examined to confirm covariate balance.

### Statistical analysis

After matching, outcomes were evaluated using risk ratios with 95% CIs. Because standard chi-square tests assume independent observations, we conducted sensitivity analyses using variance estimates clustered on matched sets. Results were materially unchanged. The assumption of independence within matched sets is acknowledged as a methodological limitation.

Additional descriptive statistics, including weighted national estimates, standard errors, and confidence intervals, were reviewed to ensure robustness and interpretability of the findings.

### Missing data

A complete-case approach was used. Records with missing essential variables were excluded during cohort construction, and the number of exclusions was negligible relative to the overall sample size.

### Ethical aspects

Because the NIS is a publicly available, de-identified database, this study was considered exempt from institutional review board (IRB) approval and did not require informed consent.

## Results

Table 1 summarizes the demographic and clinical characteristics of all 1,957,284 total THA patients, of whom 2,730 (0.1%) were dependent on renal dialysis. Patients with dialysis dependence were slightly younger than those without (64.4 vs. 66.2 years, *p* < 0.01) and had a markedly lower proportion of female patients (40.0% vs. 56.3%, *p* < 0.01).

**Table 1:** Clinical characteristics of patients undergoing total hip arthroplasty with and without renal dialysis dependence.

Parameter	No renal dialysis dependence	Renal dialysis dependence	Significance
Total surgeries	1,954,554 (99.9%)	2,730 (0.1%)	—
Average age (years)	66.2	64.4	*P <* 0.01
Female (%)	56.3	40.0	*P <* 0.01
Dyslipidemia (%)	43.7	51.3	*P <* 0.01
Chronic anemia (%)	5.6	13.7	*P <* 0.01
Osteoporosis (%)	4.6	5.1	*P <* 0.01
Type 2 diabetes (%)	15.6	43.8	*P =* 0.15
Congestive heart failure (%)	1.2	7.1	*P <* 0.01
Chronic lung disease (%)	6.6	11.5	*P <* 0.01
Liver disease (%)	1.2	1.8	*P <* 0.01
History of myocardial Infarction (%)	3.5	7	*P <* 0.01
History of cerebrovascular accident (%)	3.9	10.3	*P <* 0.01
Obesity (%)	24.4	24.5	*P =* 0.86

Several comorbidities were significantly more prevalent in dialysis-dependent patients, including dyslipidemia (51.3% vs. 43.7%), chronic anemia (13.7% vs. 5.6%), congestive heart failure (7.1% vs. 1.2%), chronic lung disease (11.5% vs. 6.6%), liver disease (1.8% vs. 1.2%), history of myocardial infarction (7.0% vs. 3.5%) and cerebrovascular accident (10.3% vs. 3.9%) (*p* < 0.01 for all).

There were no significant differences between the groups regarding obesity (24.5% vs. 24.4%, *p* = 0.86).

### In-Hospital mortality in patients with and without renal dialysis dependence in total hip arthroplasty

Table 2 presents in-hospital mortality rates for patients undergoing THA, stratified by renal dialysis dependence. Mortality was markedly higher among dialysis-dependent patients (1.30%) compared with those without dialysis dependence (0.03%), corresponding to a 31.9-fold increased risk (95% CI: 22.7–44.9, *p* < 0.01). This represents the crude (unmatched) mortality difference. The matched mortality effect size is presented in the postoperative complications analysis (RR: 10.1, 95% CI: 6.3–16.2), reflecting the adjusted risk after balancing baseline covariates.

**Table 2:** In-hospital mortality among patients undergoing total hip arthroplasty with and without renal dialysis dependence.

Parameter	No renal dialysis dependence	Renal dialysis dependence	Risk ratio (95% CI)	Significance
Died during hospitalization in %	0.03%	1.30%	31.9 (95% CI: 22.7–44.9)	*P <* 0.01

### Demographic and clinical characteristics after 10:1 propensity score matching among patients undergoing total hip arthroplasty with and without renal dialysis dependence

To mitigate confounding and reduce baseline imbalances, a 10:1 propensity score matching procedure was applied using demographic and clinical covariates including age, sex, and major comorbid conditions.

Following matching, the two cohorts were closely aligned across all measured variables (Table 3). There were no significant differences in age (64.4 vs. 64.8 years, *p* = 0.11), sex distribution (40.0% vs. 38.4% female, *p* = 0.08), or prevalence of key comorbidities such as dyslipidemia, chronic anemia, type 2 diabetes, congestive heart failure, chronic lung disease, and liver disease (*p* > 0.05 for all comparisons). Rates of prior myocardial infarction, cerebrovascular accident, osteoporosis, and obesity were also comparable.

**Table 3:** Demographic and clinical characteristics after 10:1 propensity score matching among patients undergoing total hip arthroplasty with and without renal dialysis dependence.

Parameter	No renal dialysis dependence	Renal dialysis dependence	Significance
Total surgeries	27,299 (90.9%)	2,730 (9.1%)	—
Average age (years)	64.8	64.4	*P =* 0.11
Female (%)	38.4	40.0	*P =* 0.08
Dyslipidemia (%)	52	51.3	*P =* 0.45
Chronic anemia (%)	12.8	13.7	*P =* 0.17
Osteoporosis (%)	5	5.1	*P =* 0.74
Type 2 diabetes (%)	43.3	43.8	*P =* 0.18
Congestive heart failure (%)	6.7	7.1	*P =* 0.43
Chronic Lung disease (%)	11.3	11.5	*P =* 0.74
Liver disease (%)	1.8	1.8	*P =* 0.94
History of myocardial infarction (%)	6.6	7	*P =* 0.51
History of Cerebrovascular accident (%)	11.1	10.3	*P =* 0.09
Obesity (%)	24.5	24.5	*P =* 0.98

This high degree of covariate balance supports the validity of subsequent outcome analyses, minimizing residual confounding and allowing for a more accurate assessment of the impact of renal dialysis dependence on perioperative outcomes.

### Post-matching comparison of hospital resource utilization

Table 4 summarizes hospital resource utilization after 10:1 propensity score matching. Dialysis-dependent patients had a significantly prolonged length of stay compared to matched controls (3.9 ± 3.4 vs. 2.4 ± 1.7 days, *p* < 0.01), representing a 62.5% increase.

**Table 4:** Post-matching comparison of hospital resource utilization.

Parameter	No renal dialysis dependence	Renal dialysis dependence	Percent increase	Significance
Length of stay mean in days	2.4 (Std. deviation 1.7)	3.9 (Std. deviation 3.4)	62.5%	*P <* 0.01
Total charges mean in $	60,741 (Std. Deviation 33,418)	98,454 (Std. deviation 118,945)	95.8%	*P <* 0.01

Similarly, mean total hospitalization charges were markedly higher in the dialysis group ($98,454 ± 118,945 vs. $60,741 ± 33,418, *p* < 0.01), reflecting a 95.8% cost increase despite baseline covariate balance.

These findings suggest that even after accounting for differences in patient characteristics, renal dialysis dependence is independently associated with greater hospital resource consumption during THA admissions.

### Postoperative complications after 10:1 propensity score matching among patients undergoing total hip arthroplasty with and without renal dialysis dependence

**Figure 1:**
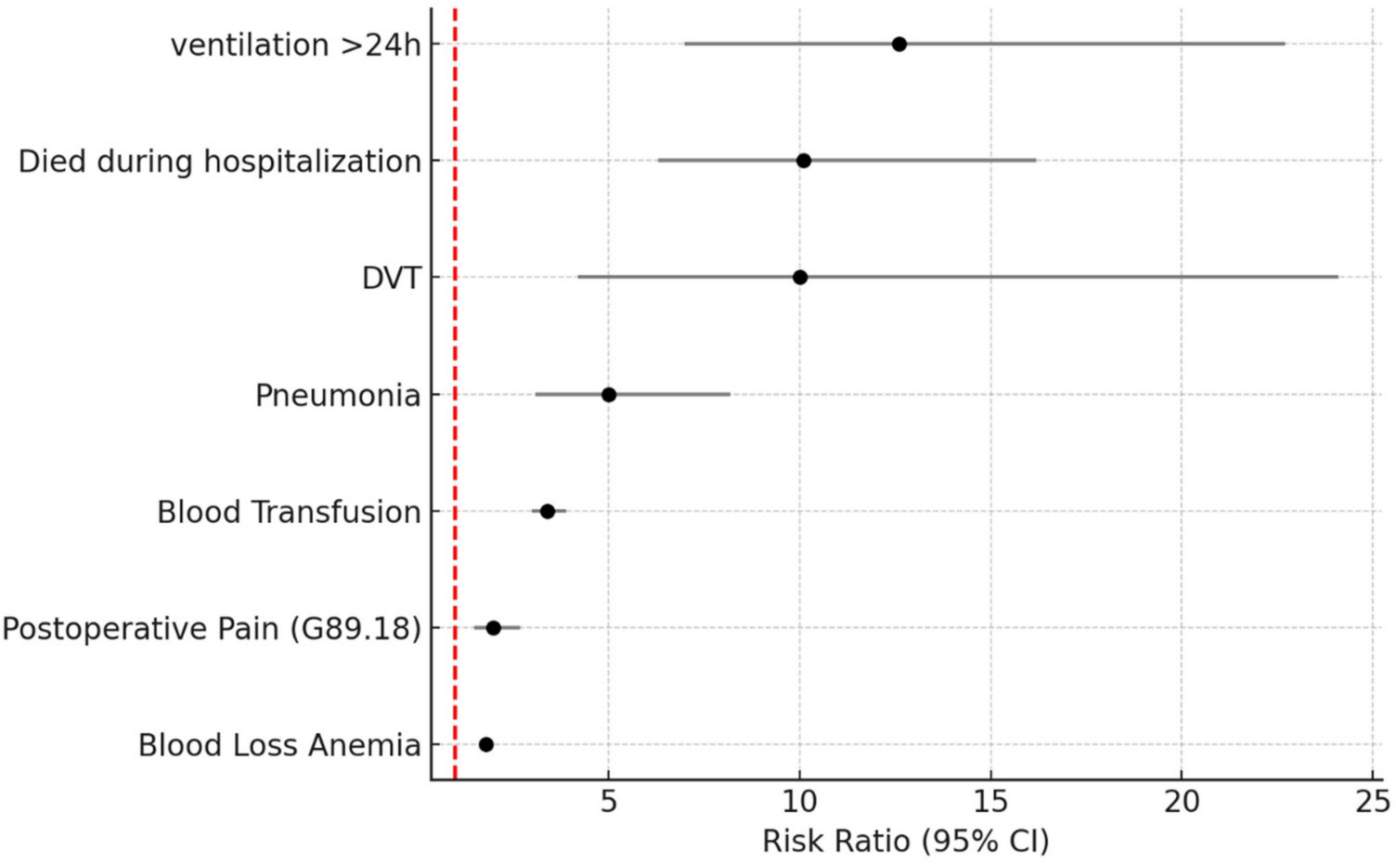
Forest plot of postoperative complications after 10:1 propensity score matching Among patients undergoing total Hip arthroplasty With and without renal dialysis dependence.

Figure 1 the risk ratios (RR) and 95% confidence intervals (CI) for major postoperative complications associated with renal dialysis dependence. Patients on dialysis had a 1.8-fold higher risk of blood loss anemia (RR: 1.8, 95% CI: 1.7–2.0, *p* < 0.01) and a 3.4-fold higher risk of requiring blood transfusion (RR: 3.4, 95% CI: 3.0–3.9, *p* < 0.01) compared with non-dialysis patients. The risk of pneumonia was also markedly elevated (RR: 5.0, 95% CI: 3.1–8.2, *p* < 0.01).

Severe complications showed even stronger associations: in-hospital mortality was 10.1 times higher (RR: 10.1, 95% CI: 6.3–16.2, *p* < 0.01), and the risk of requiring mechanical ventilation >24 h was the highest observed, 12.6-fold greater than controls (95% CI: 7.0–22.7, *p* < 0.01).

The incidence of deep vein thrombosis (DVT) was also significantly elevated (RR: 10.0, 95% CI: 4.2–24.1, *p* < 0.01), while postoperative pain was doubled (RR: 2.0, 95% CI: 1.5–2.7, *p* < 0.01). As expected, the magnitude of the mortality association decreased after matching (RR: 10.1) compared with the crude estimate (RR: 31.9), consistent with improved covariate balance.

## Discussion

In this large, nationally representative study of nearly two million primary THA patients, renal dialysis dependence was strongly associated with increased perioperative morbidity, mortality, and resource utilization. Even after rigorous 10:1 propensity score matching, dialysis-dependent patients demonstrated >60% longer hospital stays, nearly doubled hospital charges, and a markedly elevated in-hospital mortality risk (10.1-fold after matching), consistent in direction with the larger crude difference observed before matching (31.9-fold). This degree of excess risk far exceeds what is typically observed for other single comorbidities and highlights the substantial vulnerability of this patient population undergoing elective THA (4, 8).

### Interpretation of key findings

Our results highlight several clinically meaningful observations. First, dialysis patients undergoing THA had a dramatically higher burden of cardiovascular and hematologic disease, including anemia, congestive heart failure, and prior myocardial infarction, which are well-established predictors of perioperative complications (3). These comorbidities likely contribute to the increased rates of pneumonia, prolonged mechanical ventilation, and blood transfusion observed in this cohort.

Second, the magnitude of risk for hard endpoints was striking: after matching, dialysis-dependent patients had a 10.1-fold higher mortality risk and >12-fold higher need for prolonged ventilation compared with non-dialysis patients. These findings underscore the vulnerability of this population and suggest that standard perioperative protocols may be insufficient to mitigate risk. Dialysis dependence is not merely a marker of comorbidity it is a clinical state characterized by impaired host defense, altered hemodynamics, and metabolic fragility that collectively amplify perioperative hazards.

### Comparison to previous literature

Prior work examining THA in dialysis patients has been limited to small single-center cohorts or combined analyses of hip and knee arthroplasty, leading to inconsistent results. Several studies have reported elevated infection rates, increased medical complications, and early mortality among ESRD patients, but few have quantified these risks in a nationally representative and THA-specific cohort. Our study builds on this literature by offering the largest adjusted analysis to date, using modern data and robust matching methodology, thereby providing definitive evidence of the heightened perioperative risk profile for dialysis-dependent patients undergoing THA.

Our findings are consistent with registry-based analyses demonstrating excess complications and poorer implant survivorship in ESRD patients, but they further quantify the economic burden, which has received less attention in the literature. The nearly 96% increase in hospitalization costs has significant implications for bundled payment models and value-based care initiatives (9, 10).

### Clinical implications

These results carry immediate relevance for surgical decision-making and perioperative care pathways. Patient counseling should explicitly communicate the elevated risks of complications and death, allowing patients and families to make informed choices. Multidisciplinary preoperative planning including optimization of anemia, nutrition, and cardiovascular status should be considered mandatory. Coordination of dialysis timing to minimize perioperative fluid and electrolyte shifts, along with early involvement of nephrology and anesthesia teams, may mitigate some of the observed risk.

Enhanced recovery after surgery (ERAS) protocols should be specifically tailored to this high-risk subgroup, emphasizing infection prevention, early mobilization, and careful hemodynamic management. Given the high resource utilization demonstrated here, health systems and payers may consider implementing specialized perioperative pathways or postoperative monitoring programs for dialysis patients to improve outcomes and control costs (9, 10).

### Strengths and limitations

This study's strengths include its large, nationally representative sample size, ability to generate generalizable risk estimates, and use of propensity score matching to reduce baseline differences and strengthen causal inference. However, some limitations are inherent to the use of administrative databases. The NIS does not include granular clinical details such as dialysis modality, timing relative to surgery, laboratory values (e.g., hemoglobin, electrolytes), or long-term outcomes such as 30-day readmission or prosthetic joint infection. Residual unmeasured confounding remains possible despite matching (5, 11–13). Nonetheless, the consistency and magnitude of the associations observed strongly support the robustness of our finding.

Because the NIS is a stratified, weighted database, survey weights, strata, and hospital-level clustering were applied according to HCUP recommendations. Post-matching analyses assume independence within matched sets; although sensitivity checks using clustered variance estimates yielded similar results, some underestimation of uncertainty remains possible. Dialysis dependence was identified using ICD-10-CM Z99.2, which may not capture all ESRD patients or distinguish chronic from acute dialysis, introducing potential exposure misclassification. Important clinical factors such as frailty, functional status, dialysis modality, and timing of dialysis relative to surgery are unavailable in the NIS and may contribute to residual unmeasured confounding.

### Future directions

Future prospective studies should evaluate strategies to reduce perioperative risk in this population, including prehabilitation programs, optimized dialysis scheduling, perioperative erythropoietin and iron therapy, and novel blood management strategies (14–18). Given the substantial economic burden, cost-effectiveness analyses of intensified preoperative optimization protocols in dialysis patients undergoing THA are warranted.

## Conclusions

Renal dialysis dependence is a strong independent predictor of adverse outcomes following primary THA. These patients face dramatically higher mortality, complication rates, and resource use, even after adjustment for comorbidities. Our findings emphasize the need for personalized perioperative pathways, meticulous patient selection, and targeted quality improvement efforts aimed at reducing morbidity and optimizing outcomes in this high-risk population.

## Data Availability

Publicly available datasets were analyzed in this study. This data can be found at the HCUP NIS Official website. Further inquiries can be directed to the corresponding author(s).
